# Utility of 3D printed models as adjunct in acetabular fracture teaching for Orthopaedic trainees

**DOI:** 10.1186/s12909-022-03621-2

**Published:** 2022-08-02

**Authors:** S Goyal, CXK Chua, YS Chen, D Murphy, GK O.’Neill

**Affiliations:** 1 Department of Orthopaedics, University Orthopaedics and Hand & Reconstructive Microsurgery Centre, National University Health System, Level 11, Tower Block, 1E Kent Ridge Road, Singapore, 119228 Singapore; 2 Department of Orthopaedic Surgery, Ng Teng Fong General Hospital, 1 Jurong East Street 21, Singapore, 609606 Singapore

**Keywords:** Acetabular fracture, 3D printed model, Orthopaedic trainees, Teaching and assessment tool

## Abstract

**Objective:**

To evaluate the use of 3-D printed models as compared to didactic lectures in the teaching of acetabular fractures for Orthopaedic trainees.

**Methods:**

This was a randomised prospective study conducted in a tertiary hospital setting which consisted of 16 Orthopaedic residents. Ten different cases of acetabular fracture patterns were identified and printed as 3-D models. The baseline knowledge of orthopaedic residents regarding acetabular fracture classification and surgical approach was determined by an x-ray based pre-test. Trainees were then randomly assigned into two groups. Group I received only lectures. Group II were additionally provided with 3-D printed models during the lecture. Participants were then assessed for comprehension and retention of teaching.

**Results:**

Sixteen trainees participated in the trial. Both Group 1 and 2 improved post teaching with a mean score of 2.5 and 1.9 to 4.4 and 6 out of 10 respectively. The post test score for fracture classification and surgical approach were significantly higher for 3-D model group (*p *< 0.05). Trainees felt that the physical characteristics of the 3-D models were a good representation of acetabular fracture configuration, and should be used routinely for teaching and surgical planning.

**Conclusion:**

3-D printed model of real clinical cases have significant educational impact compared to lecture-based learning towards improving young trainees’ understanding of complex acetabular fractures.

**Supplementary Information:**

The online version contains supplementary material available at 10.1186/s12909-022-03621-2.

## Introduction

Anatomy education has been an essential requirement medical curriculum and the foundational pillar in surgery [1]. Didactic teaching and cadaveric models was and still is a core modality in teaching gross anatomy. As teaching pedagogy develops, many curriculums adopt a multimodal approach to include team-based interaction, problem based and simulation learning with better knowledge retention and learner experience [1, 2]. However, these methods may not directly translate to surgical residents where the problem requires a sole individual’s adept ability to visualise in three-dimensional (3D) in both clinical diagnosis, operative planning and execution.

Acetabular fractures hold a high diagnostic and surgical complexity as it can involve multiple fracture lines across the three-dimensional plane. The most common classification system is that of Judet and Letournel, which separates fractures into the 10 most common patterns, five elementary and five associated patterns [3, 4]. It relies on plain radiographs to classify the fracture pattern and whilst there is high intra- and interobserver reliability in experienced orthoapedic surgeons, less experienced surgeons and trainees may have difficulty [3, 4]. Modern technology like computed tomography (CT) and 3D reconstruction technology has provided a better understanding of acetabular fracture patterns which aids in evaluating acetabular fractures and the choice of surgical approach [4, 5]. The decision on which surgical approach in acetabulum fixation is based on accurate identification fracture pattern. Two traditional approaches are the Kocher-Langenbeck and the ilioinguinal approach. The Kocher-Lagenbeck approach is used for the posterior approach to acetabulum, while the ilioinguinal, iliofemoral or modified Stoppa approach is preferred for the anterior approach to the acetabulum [6]. Certain fracture types require more complex or modified approaches, which are not part of the residence education program and therefore not part of this study. We also appreciate that there are novel approaches in literature but these are not the traditional approach taught to resident or expected for them to know.

**Fig. 1:**
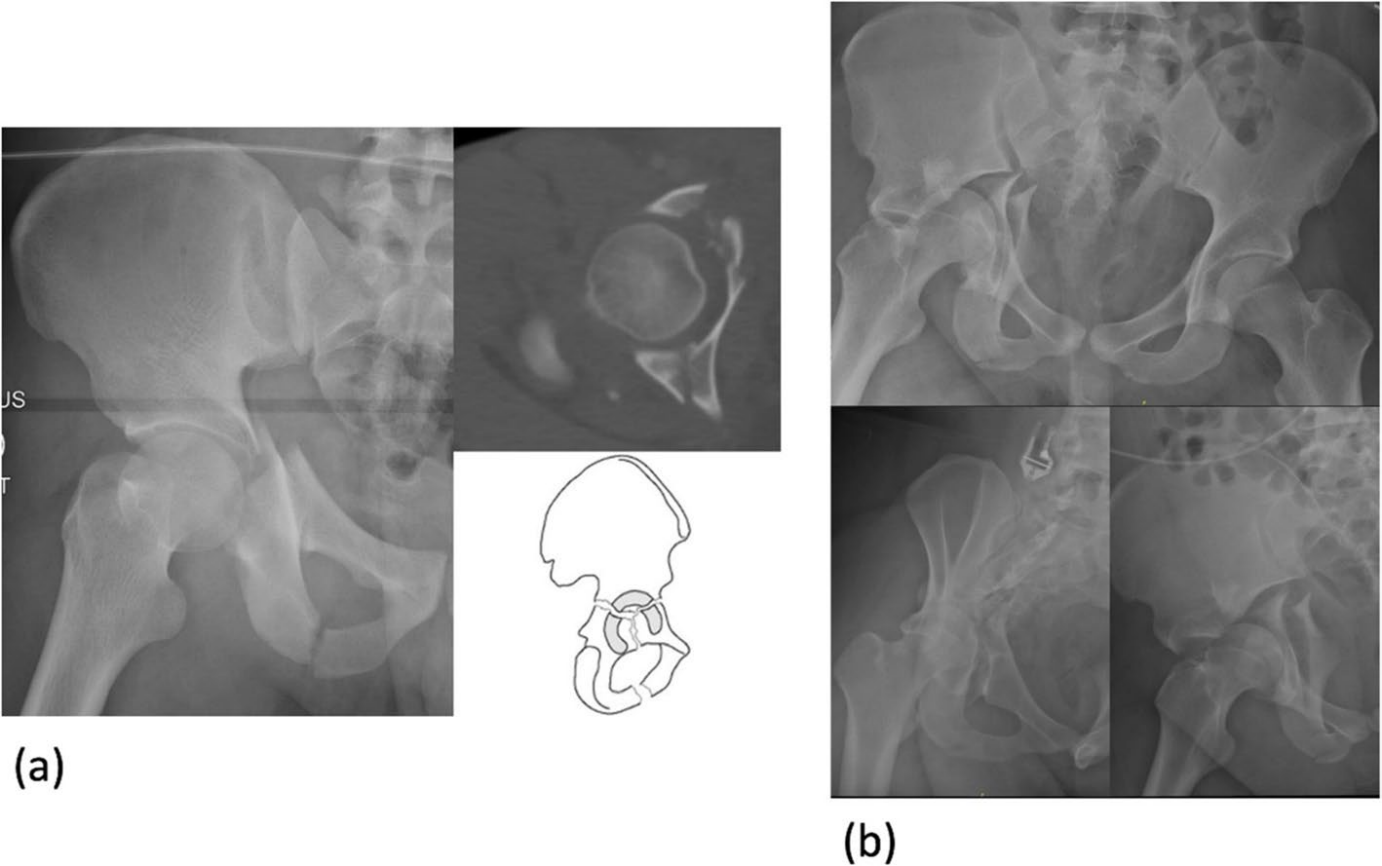
Example of ‘T-shaped’ acetabular fracture (**a**) simple textbook type used in routine teaching versus (**b**) complex pattern as seen in one of the real cases

Traditional acetabular fractures were taught through didactic lectures with 2D images (both XR and CT scan) or various algorithms and flowcharts for acetabular fracture classification [7]. However, these methods are non-intuitive and often difficult to translate into a 3D interpretation of the fracture pattern [8]. In addition, the fracture classification and textbook descriptions are generalised into the key main characteristics or patterns, however fracture lines seldom follow the classic patterns in real-life clinical scenario (Fig. 1). Besides the complexity of these fractures, its low incidence coupled with regulation of training hours result in learning opportunities becoming far and few [9, 10]. This has necessitated alternative education tools like computer-based simulator modules and other skills training models, employed in different medical speciality curriculum with varying degree of success [11].

Use of 3D technology to teach complex surgical anatomy is making its presence felt in the field of education for different surgical specialities [12–16]. 3D printed models of brain, heart, liver and complex bone structures have already shown that 3D printing of complex areas of human body can be accurate and is useful in trainee and patient education as well as for pre-operative planning and practice [17–24]. Both undergraduate and post-graduate students are favouring the use of 3D printed models as educational aids in teaching anatomy including acetabular fractures [21, 25–28].

3D printing of acetabular fractures and its application including in education has increased with improved technology, reduced cost and better availability [27, 29, 30]. These type of models have shown improved understanding of fracture patterns for trainees and possibly aid in of assessment of clinical application [23, 25, 26]. The purpose of this study is to determine the usefulness of 3D printed models of acetabular fractures as an adjunct to routine lectures for orthopaedic resident education to improve understanding of classification and assess their ability to select appropriate surgical management approach. The secondary objective of this study is to evaluate residents’ perception on the use of this novel technology.

## Methods

### Participants and study design

This randomised prospective study was approved by the National Healthcare Group (NHG) institutional review board (reference number 2017/00104) and supported by internal department funds through the ‘4^th^ Orthopaedic Research Fund Grant Call’.

The study participants consisted of 16 orthopaedic residents from our university training program. During residency a trainee spends 25% of prescribed orthopaedic training time (4 out of 16 rotations) in musculoskeletal trauma. Routine educational methods include apprenticeship model of learning combined with regular lectures, hands-on surgical training and simulator-based learning. For this study, the participants were randomised into 2 groups after obtaining written informed consent.

Participants in Group 1 were the control group who received only traditional didactic lecture via power-point slides including the basics of acetabular fracture anatomy, classification scheme and algorithms, surgical approaches and treatment using relevant diagrams and radiological imaging. The participants in Group 2 were given the 3D printed models of acetabular fractures during the teaching lecture to physically handle the pelvic models and appreciate each fracture pattern. Judet and Letournel classified acetabular fractures in 5 elementary and 5 associated fracture types [31]. All the fractures were taught to residents during the lecture using pertinent radiological images (X-ray and CT scan without 3D reconstruction) of real unidentified patients similar to what they would encounter in clinical practice and during assessments or examinations.

Before the lectures, both groups completed an x-ray based pre-test of 10 cases to assess baseline knowledge of the topic. Appropriate radiographs were shown for 10 different fracture types and participants had to comment on the fracture classification and preferred surgical approach. This was followed by segregating the groups into 2 different rooms. Subsequently, a 45-min lecture was given to each group one after the other, the difference being that of exposure of Group 2 to the 3D printed models during the lecture. To avoid immediate recall bias the post-test similar to pre-test was conducted 3 weeks after to assess the learners' retention of knowledge and understanding of the topic. This followed a feedback survey and comments on the teaching method. Responses were rated on a 5 point Likert scale from "1—strongly agree, 2—agree, 3—neutral, 4- disagree to 5—strongly disagree". The models were then given to all participants to handle and subjective feedback of individuals and the groups’ perceptions on the 3D models were also obtained.

### Patient and public involvement

No patients involved.

### Case selection for the study and printing of 3D models

Ten cases were selected from the pelvis and acetabular fracture registry of our hospital by a fellowship-trained surgeon that could represent the described patterns of acetabular fracture. The images were de-identified and subsequently analysed by two senior orthopaedic trauma specialists who routinely manage acetabular trauma and are designated faculty for postgraduate teaching. They discussed and agreed on appropriateness of each image in terms of adequate fracture pattern representation that is necessary for junior trainees to diagnose and classify the fracture. Both senior surgeons also conducted discussions and agreed on a single preferred correct answer for test questions on fracture classification and choice of appropriate surgical approach. The same cases were also used for printing the 3D models which were examined by the senior surgeons for fidelity to images and fracture types.

**Fig. 2:**
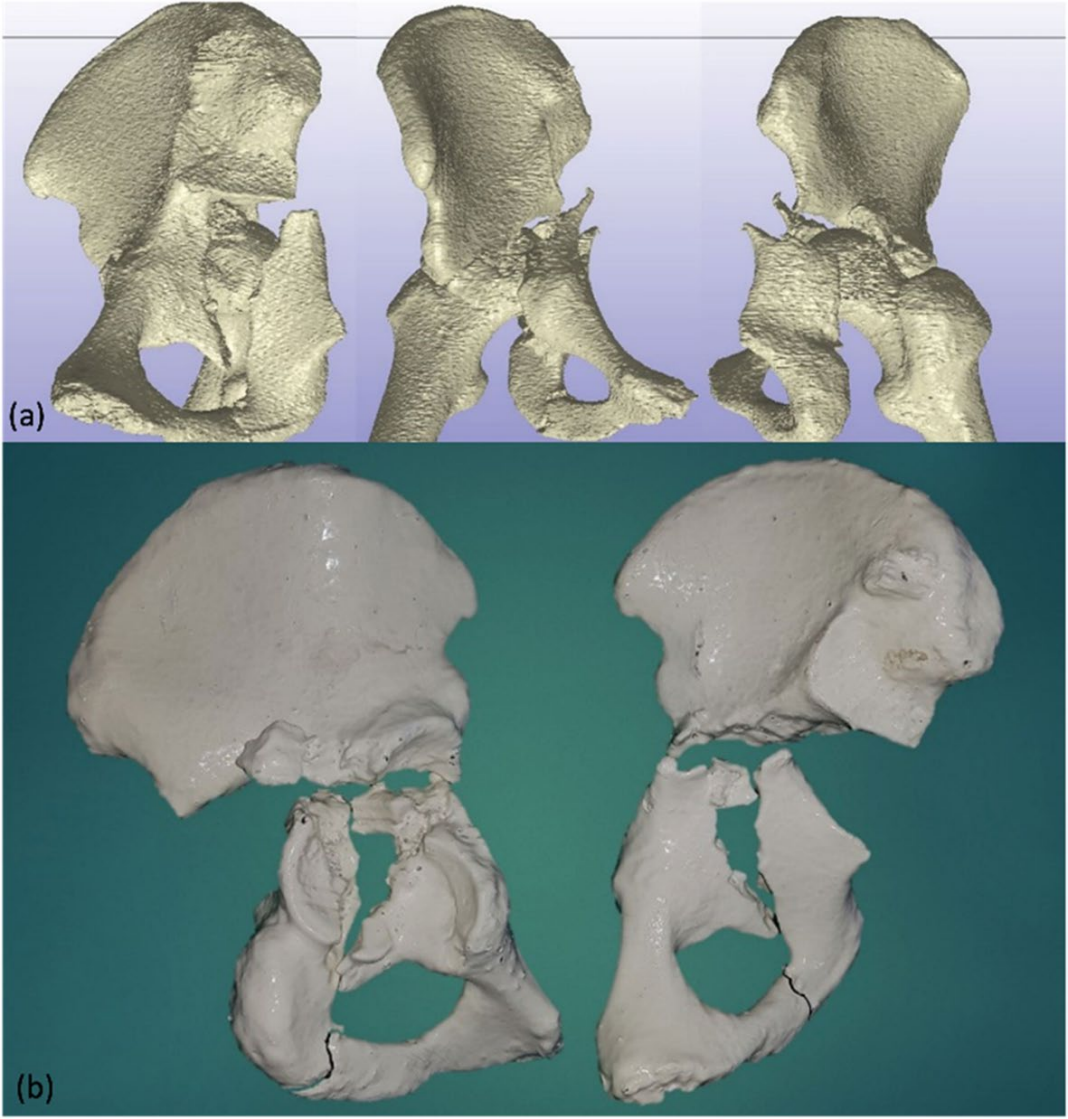
**a** Reconstruction of images from the CT scan of case of ‘T-shaped’ acetabular fracture and (**b**) 3D printed models of the same demonstrating the orientation of the fracture

Different methods and technique are available for 3D printing of medical models. For this study, the anonymized CT scans with fine-cut (< 1 mm) resolution of the selected cases were downloaded as DICOM images and outsourced to a third party company for 3D printing. They reconstructed the computer generated 3D model via a custom built SLA 3D printer using photopolymer material. Processing was performed using the ScanIP (synopsis) software and the photopolymer models were printed using custom-built SLA (Stereolithography) 3D printer (Fig. 2) with each model costing between US$ 350 – 400.

### Statistical analysis

The data was analysed using Microsoft Excel 2013 and SPSS (Ver 20.0, IBM Corp. NY, USA). The homogeneity of data was assessed and Wilcoxon signed rank test was used to determine differences in pre and post-test scores for the two groups. Post-hoc analysis could not be performed as comparative numbers in some groups was less than three. The difference is considered to be statistically significant if p-value was < 0.05. Qualitative feedback is represented as a median score and percentage of participants favouring a response. The participants’ subjective perceptions and feedback are also included in the results.

## Results

There were 16 participants with 12 male and 4 females. In total, 9 junior (years 1 to 3) and 7 senior (years 3 to 6) residents were included and randomised into two groups matched in age, and years of experience (Table 1). Both, Group 1 (Conventional lecture group) and Group 2 (3D model group) had 8 residents each.

**Table 1:** Study participants characteristics

	**Group 1 (*n* = 8)**	**Group 2 (*n* = 8)**	**Total (*n* = 16)**
Age (Range in years, mean)	30—36 (34.1)	28—37 (31.7)	28 – 37 (32.8) *p* = *0.19*
Sex (M:F)	7:1	5:3	12:4
JR: SR ratio	4:4	5:3	9:7
PG year experience (mean)	6.88	4.38	5.62 *p* = *0.15*

*JR* Junior residents (year 1 to 3), *SR* Senior resident (years 4 to 6), *PG* Post graduate

As expected, higher post-test scores were recorded compared to pre-test scores both for fracture classification with improvement to mean score of 5.2 from 2.2 (*p* = 0.001) and for correct surgical approach from the mean score of 5.1 to 6.9 (*p* = 0.004). Although the scores in diagnosis improved statistically (*p* = 0.028) for Group 1, the mean score remained below 50% (4.8 out of 10) even after teaching. The scores for correct surgical approach among Group 1 did not show a significant improvement (*p* = 0.170). For group 2, the mean post-test score not only improved to 60% (6 out of 10) and 80% (8 out of 10) for correct fracture classification and surgical approach it was also statistically significant for both variables (*p* = 0.011 and *p* = 0.016 respectively) (Table 2).

**Table 2:** Comparison of pre and post-test scores of participants (comparison of means, median)

	**Pre-test score** **(Fracture classification)**	**Post-test score** **(Fracture classification)**	***p* value**	**Pre-test score** **(Surgical approach**	**Post-test score** **(Surgical approach)**	***p* value**
Group 1—lecture group (mean ± SD, median)	2.5 ± 1.85 2.5	4.4 ± 1.91 4.7	0.03	4.8 ± 2.90 5.0	5.8 ± 1.80 5.5	0.17
Group 2—3D model group (mean ± SD, median)	1.9 ± 1.64 1.5	6.0 ± 1.90 4.5	0.01	5.2 ± 2.05 6.0	8.0 ± 1.51 8.0	0.01
Total (mean ± SD, median)	2.2 ± 1.72 2.0	5.2 ± 2.1 5.5	0.001	5.1 ± 2.43 5.5	6.9 ± 1.94 7.5	0.004

The transverse and both column fracture patterns were easiest to diagnose. The number of candidates who could correctly identify each fracture type in post-test was higher in the 3D model group when compared to the conventional lecture group especially for more complex 'associated' fractures (Fig. 2). Posterior column fracture diagnosis improved from 0 in either group to 50% (4 out of 8) and 75% (6 out of 8) in respective groups (Fig. 2). When clubbed into 'elementary' and 'associated' fractures for analysis, 40% improvement incorrect diagnosis was seen for group 2 (Table 3).

**Table 3:** Comparison for improvement in correct identification of fracture type during the pre-test and post-test between groups

	**Elementary fractures (5 simple)**			**Associated fractures (5 complex)**		
	Pre-test	Post-test	% improvement (*p* value)	Pre-test	Post-test	% improvement (*p* value)
**Group 1** (*n* = 40*)	11	19	20% (*p* = 0.10)	8	16	20% (*p* = 0.04)
**Group 2** (*n* = 40*)	13	29	40% (*p* = 0.04)	10	24	35% (*p* = 0.03)

^*^ Elementary and Associated types have 5 subtypes each; ref footnote Fig. 2. Thus, for 8 participants in each group (*n* = 8) the total correct answers = 40 (5 × 8)

**Fig. 3:**
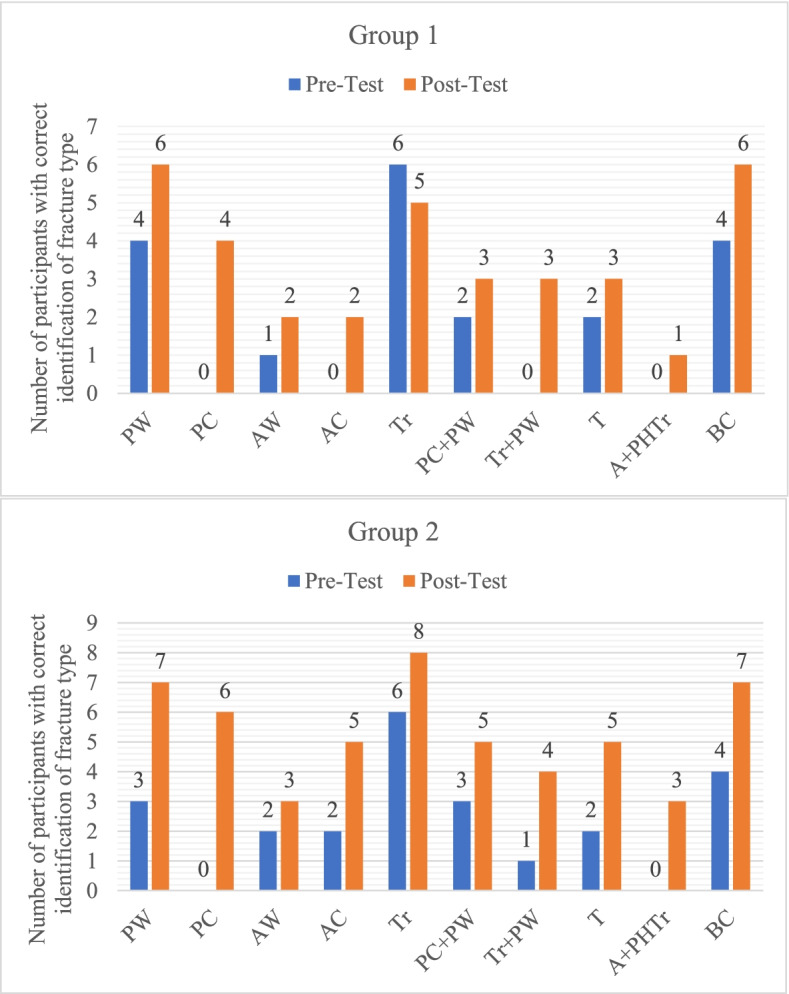
Comparison of number of participants in each group who were correctly able to identify each fracture type during the pre-test and post-test: Elementary fractures (PW – posterior wall, PC – posterior column, AW – anterior wall, AC – anterior column, Tr—transverse). Associated fractures (PC + PW – posterior column with posterior wall, TR + PW – transverse with posterior wall, T – T shaped, A + PHTr – Anterior with posterior hemitransverse, BC – both column)

**Fig. 4:**
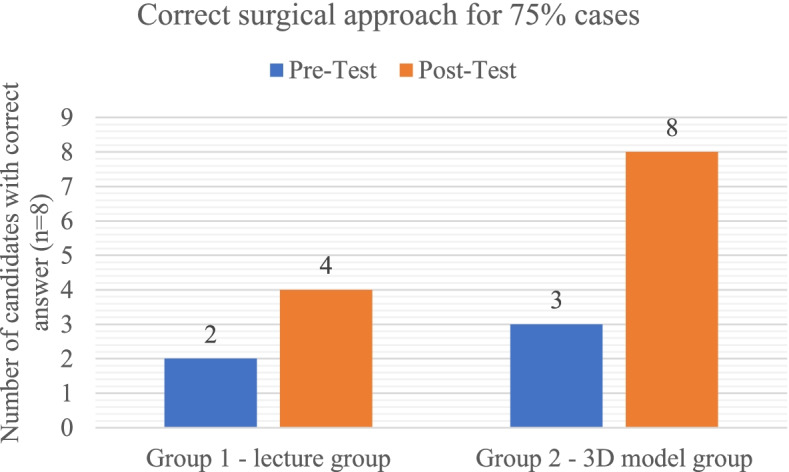
Proficiency comparison between groups for correct identification of surgical approach in 75% of cases

The ability to plan the correct surgical approach depends not only on the correct identification of fracture but understanding the fracture pattern. Candidates were deemed proficient if they could propose a correct surgical approach for least 75% of cases; which in our study was considered as of 7 out of 10 cases. Fifty per cent (4 out of 8) in Group 1 showed this proficiency compared to 100% (8 out of 8) in Group 2 after exposure to the teaching method (Fig. 3 and 4).

Participants were asked to provide perceptive feedback on their teaching method based on a 'Likert' scale from 1 to 5 (1 – strongly agree, 2 agree, 3 – neutral, 4 – disagree and 5 – strongly disagree) (Table 4).

**Table 4:** Perceptive feedback of participants to their teaching method

**No**	**Feedback questions**	**Group 1—lecture group** **(median)**	**Group 2—3D model group** **(median)**	***p* value**
**1**	This method teaches clinically relevant anatomy	2	1	0.10
**2**	This method accurately depicts acetabular fracture types	2	1	0.10
**3**	This method improved 3D spatial comprehension of acetabular fractures	2	1	0.40
**4**	This method improves Xray interpretation	2	1	0.26
**5**	This method helps to improve CT scan interpretation	3	2	0.19
**6**	This method helps to better learn acetabular fracture classification	2	1	0.31
**7**	This method helps to understand surgical approach	3	1	0.05
**8**	This method helps to understand fracture reduction and surgical fixation	3	1	0.12
**9**	I would use this method to prepare for acetabular surgery	2	1	0.10
**10**	This method should be extended to teach other fractures	3	1	0.03

based on ‘likert’ scale of 1–5 (1 – strongly agree, 2 – agree, 3 – neutral, 4 – disagree and 5 – strongly disagree)

Although both groups responded favourably to their teaching method, residents in Group 1 were unsure of using lecture teaching for practical application (Table 4). Group 2 strongly agreed that the 3D models helped in accurate diagnosis and classification, surgical approach and planning, while also appreciating the perceived need for 3D models in teaching other fractures as well. Six out of eight (75%) participants strongly agreed while other 2 agreed that they preferred 3D models to be incorporated in regular curriculum for teaching acetabular fractures. In subjective feedback from Group 1, six participants said that did not prefer the lecture-based teaching after they were provided with the 3D models to play with upon completion of the study. Some comments from the participants in favour of the 3D printed models were –

- "Models improved my 3D understanding of fracture lines and patterns seen on X-ray"
- "Lectures can become confusing but the models allow for simultaneous correlation to images on X-ray and CT scan"
- "I am now more confident in the diagnosis and classification of acetabular fractures"

## Discussion

The management of acetabular fractures is a subject of interest for trauma surgeons and orthopaedic residents often find them daunting to understand [25, 26]. Classification of these fractures are complex and it also dictates the surgical approach to take, hence it is important for orthopaedic residents to have an adept understanding and our study. The 16 orthopaedic trainees demonstrated this difficulty with only correctly diagnosis an average of 2 out of the 10 fracture types in the pre-test. After the teaching session, not only did they correctly diagnose more than 50% cases correctly the feedback responses also showed that all residents felt more confident about acetabular fractures.

Efforts have been made in the past to introduce algorithms for classification of acetabular fractures [7]. In our study, we compared the conventional lecture-based teaching method using these algorithms to the same lecture augmented with 3D models and the use of 3D models showed improvements in learners' ability to classify fracture and propose suitable surgical approach. Few studies have employed a similar model but have either used artificially created fracture or limited fracture types [25, 26, 32]. We obtained the clinical data and radiological imaging from actual patients as the fracture lines seldom follow the textbook description in real life. Also, we felt it is important to visualise variations in different fracture patterns, hence we made efforts to obtain all 10 fracture types described by Judet and Letournel for our residents. One can argue that when teaching or assessment of classification ability is required, the subjects should have access to all options. Awan et al. obtained models for five most common acetabular fractures whereas Lim and colleagues, in their study method, did not choose single column or wall fractures assuming that it would not adequately test the ability of 3D models to help residents with classification [25, 26]. Our study not only shows that elementary fractures were difficult to identify for residents but also that this ability improved significantly for those who had access to 3D printed models (Table 3).

Incidence of acetabular fractures is low and good surgical execution affects the functional outcome [10]. We believe that handling of 3D models while learning the descriptions on 2D images (X-ray and CT scans) helped our residents to better understand the orientation of fracture fragments in relation to its anatomic landmarks. This is shown by significant improvement number of participants who were able to provide the correct surgical approach when given 3D models to aid in learning. We also found that the use of 3D models had a greater benefit in fractures involving the posterior column or complex fractures such as an anterior with posterior hemi-transverse fracture or complex both-column fractures. This could be because that simple fracture patterns are more common and easily appreciated compared to complex acetabular fracture with fracture lines running in more than one direction and demand a higher ability in visual-spatial understanding. In addition, the availability of physical 3D models would allow them to experiment with the pieces of fracture fragments like a puzzle and appreciate different reduction manoeuvres and surgical technique. Previous authors have shown similar results of improvement in test score for correct classification of acetabular fractures but were not able to show this extension of the diagnostic knowledge to clinical application [25, 26]. Also, the study by Awan et al. was for radiological residents only where diagnosis is endpoint.

Recent advancements in computer technology have allowed for 3D modelling, this has also led to many forms of digital anatomy simulations such as virtual and augmented reality technology [33]. Users can interact with vivid imagery through head-mounted displays or desktop systems to appreciate the human anatomy without the limitations of donation shortages [34]. Although current studies suggest that virtual learning resources lack the tactile experience and cannot replace cadaver and physical models, it can be used as an adjunct to 3D models [35–37]. This could not only aid teaching of fracture patterns and classification, but also translate to pre-operative planning and practice surgery for trainees. Kim et al. and Zeng et al. have shown this is possible as per results from their respective papers [38, 39].

ACGME (Accreditation Council for Graduate Medical Education) from North America and other European medical education accreditation systems has shifted focus on developing core competencies for resident training in surgical training to assess competency is now being incorporated into the curriculum [40–42]. Our study also shows that 3D printed models can also be used to assess one of the core competencies i.e. practice-based learning. The results of our study suggest an overall deficiency in young trainee's ability to identify acetabular fractures which improved with the use of 3D printed models from real patients and also improved their application of this knowledge. We suggest that this model can also be used as an additional tool to tick the checklist of EPAs.

Feedback is an essential tool in educational methods. As much as the trainees require feedback on their performance, so does the system on the methods of teaching. Even though both groups showed a positive response to their teaching method, digital images cannot resolve the difficulty of understanding 3D orientation of fractures. After showing the 3D models to whole group, 100% participants favoured 3D printed models to only lecture-based learning. Throughout the feedback and post-study discussions, 3D models were favoured method to understand acetabular fracture anatomy, improved orientation in XR and CT images as well as a need for use in surgical planning. The models provide operating surgeon with visual and tactile model of fracture to appreciate the personality of fracture, and manoeuvre them to understand surgical reduction techniques.

### Limitations

A recent systemic review has demonstrated multiple shortcomings in studies about the use of 3D technology for the training of surgical trainees [43]. We aimed to perform a well-designed trial but the use of 3D printing technology in medical education is new and standardised assessment methods are constantly being developed. The small sample size was one of the limitations, however this was a single centre study with all participants undergoing the same training curriculum and reduces the risk of bias due to differing training experience. A multi-centre simultaneous trial with a large sample size would power the study immensely. We recognise the gender distribution between the groups were uneven, however greater emphasis was placed on trainee years and postgraduate experience compared to the role that gender may play on learning and performance [44]. Secondly, our post-test was done only once at 3 weeks from teaching and while repeated assessments over a period of time with regular use of the models would provide more accurate data on their utility as education and assessment tool. We tried to limit certain bias like pro-innovation bias in pre-test by obtaining informed consent immediately before randomisation for the lecture. Immediate recall bias was also limited by conducting the post-test after 3 weeks and avoiding interim make up lectures.

In terms of 3D models themselves, only osseous anatomy was printed in our pilot study. The use of variable materials to add on soft tissues and body surface in future would allow ‘real feel’ of the cases. This would allow candidates to be to perform ‘real’ practice surgery and has potential to develop into more sophisticated education tools. The 3D printed models can not only be used for orthopaedic trainees’ teaching and assessment of knowledge, they can be produced routinely as representative specimen of everyday complex fractures.

## Conclusion

Use of 3D printed models of acetabular fractures as an adjunct to conventional lectures to improve orthopaedic residents understanding of the classification and application to surgical planning. 3D printed models provide a tactile learning of fracture patterns variability as concluded by our trainees’ preferred method for learning. Incorporating this model to assess surgical planning can make it a useful assessment tool for educators. The new generation of surgical trainees have various modern technology at their disposal in everyday life which should be incorporated into regular training at all levels.

The field of education has suffered when it comes to innovation in teaching method and even in medical education we are only recently and using modern technologies for improving upon traditional methods like didactic lectures and the apprenticeship model. Landmark Italian educator Maria Montessori had demonstrated how powerful it can be to let children see, touch, hear, smell or taste what they are learning but the ways of early childhood teaching changed only after half a century later [45]. We are all multi-sensory learners and the introduction of multimodal techniques e.g. simulators and 3D models would enhance ones learning ability to understand complex forms and concepts in medical science.

## Supplementary Information

**Additional file 1:** **Supplementary figure 1.** Acetabular fracture Judet-Letournel classificaiton.

## Data Availability

The dataset generated and analysed during the current study are not publicly available as the data is non digital but are available from the corresponding author on reasonable request.
