# Supplementary material for: Utility of 3D printed models as adjunct in acetabular fracture teaching for Orthopaedic trainees
Source: BMC Med Educ. 2022 Aug 2;22:595. doi: 10.1186/s12909-022-03621-2 (PMC9344721; doi:10.1186/s12909-022-03621-2)
Supplement: Supplementary file 1 — Additional file 1: Supplementary figure 1. Acetabular fracture Judet-Letournel classificaiton. [file 12909_2022_3621_MOESM1_ESM.docx]

Supplementary figure 1: Acetabular fracture Judet-Letournel classificaiton


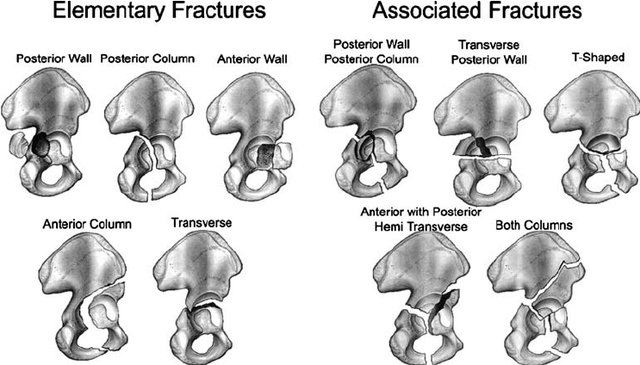


^1^ JUDET R, JUDET J, LETOURNEL E. FRACTURES OF THE ACETABULUM: CLASSIFICATION AND SURGICAL APPROACHES FOR OPEN REDUCTION. PRELIMINARY REPORT. J Bone Joint Surg Am. 1964;46:1615-1646.

^2^ Pagenkopf E, Grose A, Partal G, Helfet DL. Acetabular fractures in the elderly: treatment recommendations. HSS J. 2006;2(2):161-171.
